# Prevention and management of COVID-19 among patients with diabetes: an appraisal of the literature

**DOI:** 10.1007/s00125-020-05164-x

**Published:** 2020-05-14

**Authors:** Prasad Katulanda, Harsha A. Dissanayake, Ishara Ranathunga, Vithiya Ratnasamy, Piyumi S. A. Wijewickrama, Nilukshana Yogendranathan, Kavinga K. K. Gamage, Nipun L. de Silva, Manilka Sumanatilleke, Noel P. Somasundaram, David R. Matthews

**Affiliations:** 1 University Medical Unit, National Hospital of Sri Lanka, Colombo, Sri Lanka; 2 Diabetes Research Unit, Department of Clinical Medicine, Faculty of Medicine, University of Colombo, 25, Kynsey Road, Colombo, 00800 Sri Lanka; 3 Harris Manchester College, University of Oxford, Oxford, UK; 4 Oxford Centre for Diabetes, Endocrinology and Metabolism, University of Oxford, Churchill Hospital, Headington, Oxford, OX3 7LE UK; 5 Diabetes and Endocrine Unit, National Hospital of Sri Lanka, Colombo, Sri Lanka; 6 Department of Clinical Sciences, Faculty of Medicine, General Sir John Kotelawala Defence University, Colombo, Sri Lanka

**Keywords:** Coronavirus, COVID-19, Diabetes, SARS-CoV-2

## Abstract

The coronavirus disease 2019 (COVID-19) pandemic has emerged as one of the greatest challenges faced by humankind in the recent past. People with diabetes and related comorbidities are at increased risk of its complications and of COVID-19-related death. Older age, multi-morbidity, hyperglycaemia, cardiac injury and severe inflammatory response are predictors of poor outcome. The complex interplay between COVID-19, diabetes and the effects of related therapies is being explored. Most patients experience a mild illness with COVID-19, while people with diabetes are at increased risk of severe disease. Optimising glycaemic control and adopting measures to prevent disease spread are critical aspects. The management of mild disease is supportive, while very many immunomodulatory and antiviral therapies are being investigated for the treatment of severe disease. Several of these agents have specific considerations for use in people with diabetes. Since mass population lockdowns are considered a key step in controlling disease spread, it follows that, in addition to the direct vulnerability to severe COVID-19, people with diabetes can be affected by limited access to healthcare, insulin, other medications and blood glucose monitoring equipment. Measures to prevent disease spread at the individual and community level are the key to mitigating the rapidly escalating pandemic, while agents for chemoprophylaxis and vaccines are being explored. People with diabetes should be recognised as a vulnerable group for complicated disease and are at risk during times of disturbed social systems. Strategies are needed to safeguard the health of patients with diabetes during the pandemic. This review summarises the current knowledge and perceived challenges for prevention and management of COVID-19 in people with diabetes.

**Electronic supplementary material:**

The online version of this article (10.1007/s00125-020-05164-x) contains peer-reviewed but unedited supplementary material including a slideset of the figures for download, which is available to authorised users.

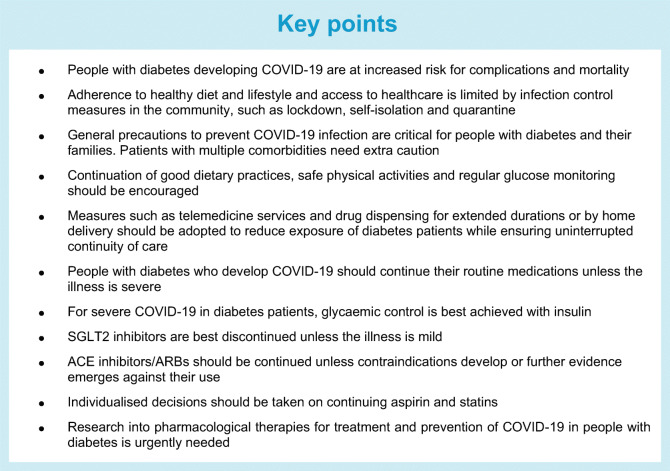

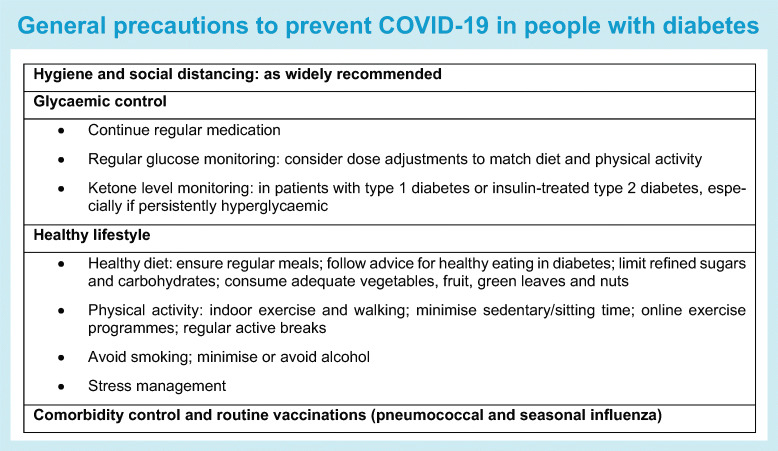

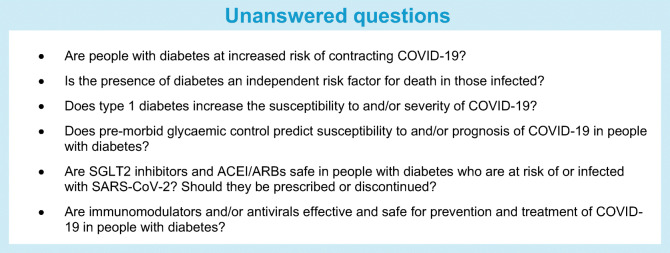







## Introduction

Coronavirus disease 2019 (COVID-19), caused by the severe acute respiratory syndrome-coronavirus-2 (SARS-CoV-2) virus, has become a pandemic within a few months after it was first described in Hubei province in China. At the time of writing it had affected over 1,800,000 individuals in more than 200 countries and territories worldwide causing over 110,000 deaths [1]. The USA and certain regions of Europe are currently experiencing the highest disease burden while it is apparently coming under control in China [2].

COVID-19 is highly transmissible from person to person through respiratory secretions. The virus enters through mucous membranes of the upper respiratory tract, later affecting lungs. [3]. In the majority of cases, COVID-19 is a mild illness, while some people develop severe disease characterised by respiratory compromise (dyspnoea; respiratory rate ≥ 30 breaths per minute; blood oxygen saturation ≤ 93%; PaO_2_:FiO_2_ < 300; and/or pulmonary infiltrates on >50% of lung fields on radiological imaging) [4]. A minority of patients develop critical disease with septic shock or respiratory and/or multi-organ failure. Fewer than 5% of those affected develop serious or critical illness [5], which is likely to be an over-estimate since sub-clinical infection rates in the community are unknown. Secondary pneumonic bacterial infection can be an additional problem.

## Diabetes and infections

Both type 1 and type 2 diabetes increase the susceptibility to infections and their complications [6]. Neutrophil dysfunction, reduced T cell response and disordered humoral immunity are contributory [7], and bacterial and viral respiratory tract infections are particularly common [8]. Diabetes is associated with increased morbidity and mortality risk from pneumonia [9], and hyperglycaemia on admission for pneumonia (>11 mmol/l) predicts poor outcome [9]. During the SARS epidemic in 2002/2003, diabetes was an independent predictor of mortality risk (OR 3.0; 95% CI 1.4, 6.3; *p* = 0.005) [10]. The presence of comorbidities, including diabetes, also increased mortality risk (independent of age) during the Middle East respiratory syndrome-coronavirus (MERS-CoV) epidemic in 2012 (adjusted HR 3.74; 95% CI 2.57, 5.67) [11]. In another study, diabetes had the strongest impact on mortality risk among MERS-CoV patients [12]. Similarly, among young patients with novel influenza A (H1N1) in 2009, diabetes increased the risk of intensive care unit (ICU) admissions (adjusted OR 4.72; 95% CI 1.81, 12.3) [13].

## COVID-19 and diabetes

### Increased risk for severe disease

According to the available evidence, people with diabetes do not have a higher susceptibility to SARS-CoV-2 infection [14]. However, observations in the recent COVID-19 pandemic are comparable to those from other epidemics, with higher rates of complications and mortality among patients with diabetes. Hypertension, diabetes, coronary artery disease and cerebrovascular disease were the main associations with severe disease (present in 23.7%, 16.2%, 5.8% and 2.3%, respectively, of people severely affected by COVID-19) [15] and mortality rate (53.8%, 42.3%, 19.2% and 15.4%, respectively, of people who died with the infection) [16]. Immunocompromised state, obesity and tobacco smoking are other risk factors for severe disease and death [3, 17, 18].

A larger study of 72,314 patients with COVID-19 in China indicated that patients with diabetes had a threefold higher mortality rate compared with the mortality rate in COVID-19 patients overall (7.3% vs 2.3%) [19]. In Italy, where the overall case fatality rate is higher (7.2%, compared with 2.3% in China), among a group of 355 COVID-19 fatalities, 35.5% had diabetes and 30% had ischaemic heart disease [20].

Older age, the presence of two or more comorbidities and obesity also predict poor prognosis among COVID-19 patients [17, 21]. These are common associations of diabetes and may contribute, at least in part, to the observed increased risk. Nevertheless, in a nationwide study of 1590 COVID-19 patients in China, after adjusting for age, smoking and comorbidities, diabetes was an independent risk factor for the composite outcome of increased ICU admission, need for ventilation and death (HR 1.59; 95% CI 1.03, 2.45; *p* = 0.037) [21]. So far, no published data are available on disease severity among younger patients with type 1 diabetes, although experts in the field have observed it to be similar to those without [22].

### Prognostic markers

Similar to previous studies among patients with influenza and bacterial pneumonia, elevated serum ferritin, lactate dehydrogenase, C-reactive protein (CRP), procalcitonin and erythrocyte sedimentation rate (ESR) predicted severe disease among patients with COVID-19 [23, 24]. This may indicate secondary bacterial infection exacerbating COVID-19. Increased serum ferritin, in particular, might suggest a severe secondary bacterial infection among these patients, thereby making it useful as a cost-effective prognostic marker [23, 24]. Lymphopaenia was also associated with very severe disease [23, 24]. Raised D-dimer levels were observed in severe illness, suggesting a possible consumptive coagulopathy [25], while anticoagulation was linked to decreased mortality rate in COVID-19 patients [26].

Among 174 COVID-19 patients in Wuhan, China, people with diabetes had a greater inflammatory response (higher CRP, ESR and IL-6, and relative neutrophilia and lymphopaenia), higher incidence of coagulopathy (higher D-dimer levels), metabolic derangements (hyperglycaemia, transaminitis), severe pneumonia (higher radiological scores) and higher mortality rate, compared with those without [27]. However, people with diabetes in this study were older and had higher prevalence of cardiovascular disease. It is noteworthy that diabetes itself is a proinflammatory and prothrombotic state [28]. The data indicate that COVID-19, at least in its severe forms, is a state of severe inflammation and thrombotic tendency, so those with diabetes may be predisposed to such intense immune dysfunction resulting in severe late disease. This is further supported by the observation that renal and cardiovascular comorbidities, which add to the proinflammatory state, further worsen the outcome [27].

Elevated N-terminal pro-brain-type natriuretic peptide (NT-proBNP) and cardiac troponin I (cTnI), were significantly correlated with severe disease, suggesting that COVID-19 may lead to myocardial injury and impair cardiac function [15]. In people with diabetes and pre-existing ischaemic heart disease, limited cardiac reserve may increase morbidity and mortality risk.

### ACE2, SARS-CoV-2 and diabetes

**Fig. 1:**
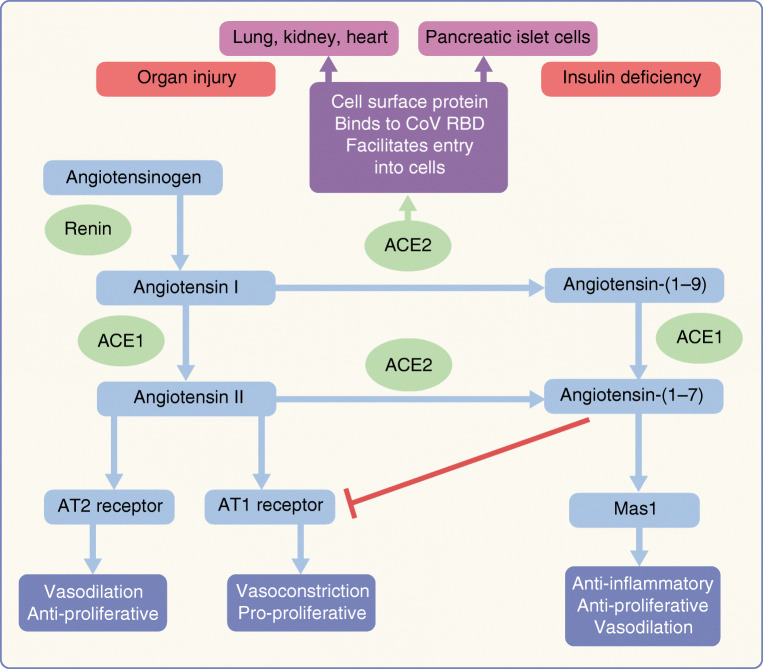
Role of ACE2 in the pathogenesis of coronavirus diseases. ACE2 converts angiotensin I and angiotensin II to angiotensin-(1–9) and angiotensin-(1–7), respectively. ACE2 is also expressed in the lung, kidney, heart and pancreas and acts as a facilitator for CoV entry into cells. Use of ACEI/ARBs increases angiotensin I levels and upregulates *ACE2* gene expression. This facilitates excess viral entry into host cells causing organ injury and insulin deficiency, contributing to hyperglycaemia. Upregulated ACE2 may convert angiotensin II to angiotensin-(1–7). The latter acts on the Mas1 receptor to trigger anti-inflammatory effects and inhibits the AT1 receptor to cause vasodilation. However, at least in ACEI users, angiotensin II levels will be low and the net benefit of ACE2 upregulation is uncertain. CoV infection downregulates ACE2 expression, thereby reducing angiotensin-(1–7) levels, which reduces its anti-inflammatory effects and potentially worsens organ vulnerability to infection. AT, angiotensin; RBD, receptor binding domain. This figure is available as part of a downloadable slideset

The receptor binding domain of SARS-CoV-2 uses host ACE2 for fusion of viral and host cell membranes [29]. ACE2 is a type 1 integral glycoprotein highly expressed in the kidney, endothelium, lungs and heart [30]. ACE2 converts angiotensin I and II to angiotensin-(1–9) and angiotensin-(1–7), respectively (Fig. 1). The latter acts as a vasodilator and has anti-inflammatory and cardioprotective effects. According to animal studies, expression of ACE2 in lung tissues is low under normal conditions [31], but is upregulated during lung injury [32]. In fact, some animal studies [33] and pilot studies in humans [34] have suggested a potential therapeutic role of ACE2 against inflammatory acute lung injury. Furthermore, SARS-CoV (the virus responsible for the 2002/2003 SARS epidemic) appears to downregulate ACE2 expression in infected cells, and this is thought to perpetuate the inflammatory injury [35].

In 2003, patients with SARS had higher rates of hyperglycaemia on admission than non-SARS pneumonia patients, irrespective of their pre-morbid glycaemic status, disease severity or glucocorticoid use [10]. Subsequently SARS-CoV was shown to bind to ACE2 in pancreatic islet cells, damage them and cause acute hyperglycaemia, possibly contributing to an excessive mortality rate, even among people without diabetes [36]. A similar mechanism may operate in SARS-CoV-2 infection, contributing to hyperglycaemia, excess complications and mortality rate.

Although ACE2 shares some characteristics with ACE1, it is not inhibited by ACE inhibitors (ACEI). In fact, ACEI and angiotensin receptor blockers (ARBs) upregulate ACE2 expression [37]. Therefore, it has been postulated that ACEI/ARB use might facilitate infection with SARS-CoV-2, resulting in severe disease [38, 39]. Furthermore, ACE2 gene polymorphism has been linked to increased risk of diabetes and cardiovascular disease and this may also predict susceptibility to severe CoV infection [39]. By contrast, upregulated ACE2 may increase levels of angiotensin-(1–7), mounting an anti-inflammatory effect. However, low angiotensin II levels (due to ACEI inhibition) may mitigate this benefit. Thus it is still unclear what the balance between benefit and risk might be in continuing or stopping ACE inhibition [32].

### Clinical presentation and diagnosis

Following an incubation period of 2–14 days (median 5 days), the majority of people with COVID-19 will present with cough, fever, shortness of breath and, less commonly, nausea and diarrhoea [40]. A late phase of sudden deterioration is observed in some patients after about 7–10 days of fever. This is characterised by sudden deterioration in oxygen saturation.

People with diabetes develop similar symptoms. However, the initial manifestation could be milder, fever may be less common (59.5% vs 83.2%; *p* = 0.02), and deterioration could occur rapidly in later stages [27]. Deteriorating glycaemic control and hyperglycaemic emergencies may be a presenting feature, and those with type 1 diabetes may present with diabetic ketoacidosis (DKA) [41].

Real-time reverse transcription PCR (rRT-PCR) remains the gold standard for diagnosis of SARS-CoV-2 infection [42]. However, its use is limited by cost, the need for technology and expertise, and limited availability in resource-poor settings. IgM-based rapid diagnostic tests and other ELISA-based serological tests are being developed. Their key limitation is poorer early sensitivity because it typically takes 5 to 7 days for patients to seroconvert [43]. Tests for viral antigen detection are also being developed.

### Management considerations

**Fig. 2:**
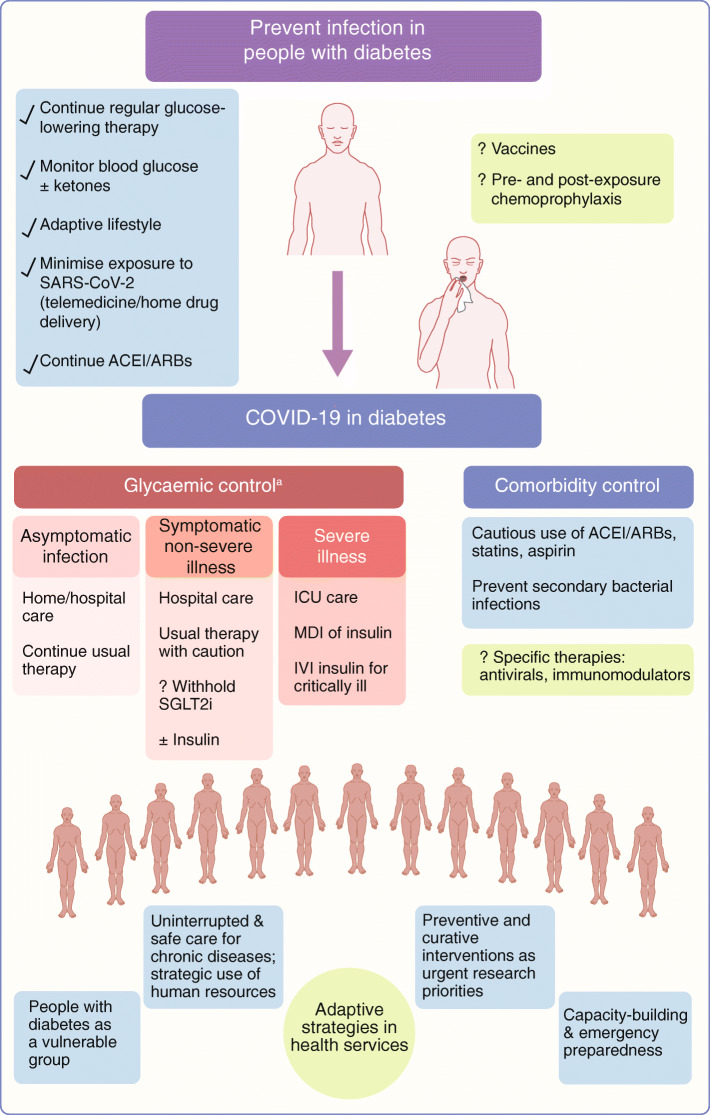
COVID-19 preventive and management considerations for people with diabetes at an individual level and from a healthcare service perspective. ^a^Owing to limited evidence, and risk of dehydration and euglycaemic ketoacidosis, it may be advisable to discontinue SGLT2 inhibitors until recovering from acute illness and/or until further evidence is available. Insulin is safe, provided glycaemic status is regularly monitored and treatment is adjusted. Other agents should be used with caution, particularly in hospitalised patients. IVI, intravenous infusion; MDI, multiple daily injections. This figure is available as part of a downloadable slideset

People with diabetes need timely integrated interventions to prevent them acquiring the disease. Enhanced self-management, supportive healthcare services and public health measures need to be in place (Fig. 2). If the illness is contracted, it should be promptly recognised and supportive measures instituted, with specific attention to glycaemic control. Further advice on this can be found in the EASD e-Learning portal: https://easd-elearning.org/covid-19/.

### Patients with diabetes at risk of COVID-19

The COVID-19 pandemic is far from being solely a medical phenomenon. It disrupts personal and professional lives severely and affects people and societies on several levels. The key strategies promoted for containment of an outbreak such as isolation, social distancing and lockdown of cities can have a significant impact on the health and lifestyle of an individual. Management of a chronic disease such as diabetes, which requires dietary modification, regular exercise and good adherence to medications, poses many complex challenges.

Maintaining a healthy diet may be challenging because of limited access to appropriate food. Careful portion selection and adjusting mealtime insulin according to carbohydrate intake continues to be the best strategy, particularly for patients on multiple-dose insulin regimens.

Adopting a regular exercise plan might not be feasible owing to social distancing, restrictions on outdoor activities and concerns over the high risk of disease spread in gyms and sports centres (many of which are closed during lockdown, depending on regional restrictions). Activities such as indoor walking, gardening and stationary high intensity activities may be suitable alternatives to maintain an active lifestyle.

Regular monitoring of blood glucose is important. Continuous glucose monitoring (CGM) and flash glucose monitoring systems are useful and allow remote monitoring by healthcare providers (however see below under ‘Management of patients with diabetes and COVID-19’ for possible concerns regarding the use of paracetamol/acetaminophen alongside CGM). For patients with type 1 diabetes, monitoring of ketone levels (particularly for people who are persistently hyperglycaemic) and vigilance for the development of symptoms of DKA are important.

It is also important that people with diabetes have an adequate supply of their medications at home. In fact, increased incidence of DKA among children with type 1 diabetes has been observed due to a delay in seeking medical treatment and in providing routine healthcare for newly developed symptoms, as patients are afraid of contracting COVID-19 from healthcare settings [22].

Routine clinic visits and overcrowding in hospitals should be minimised to reduce disease spread among people with diabetes. Measures such as telemedicine consultations or telephone advice, issue of medication to caregivers at lower risk, online coordination of drug delivery, and dispensing medications for extended periods should be considered by healthcare providers. Non-emergency routine evaluations (for foot disease, retinopathy etc.) should be deferred, as close contact between the healthcare providers and patients may lead to increased risk of COVID-19 transmission. Patients with limb-threatening or vision-threatening complications should be triaged for immediate attention.

During this time of uncertainty, fear, helplessness and strong emotions may increase stress in some patients. It is important to ensure psychological wellbeing, as stress may adversely affect glycaemic control. The WHO recommends minimising reading or listening to news that could cause distress and anxiety. Regular sleep routines are important. Relaxation techniques such as meditation can help people with stress and anxiety. Maintaining contact with relatives, friends and neighbours via telephone conversations or using online communication platforms can help to reduce the effects of social isolation [44].

Employers should consider work from home strategies, or furlough, for employees with poorly controlled diabetes or those with cardiac or renal complications, particularly for those in high risk occupations such as frontline healthcare work or similar high risk jobs, and especially in areas with high COVID-19 prevalence.

### Management of patients with diabetes and COVID-19

#### Setting and general considerations

The majority of people with COVID-19 will develop a mild disease that can be managed at home according to local guidelines. Patients with diabetes developing symptoms suggestive of COVID-19 infection should immediately notify local healthcare services to determine the need for diagnostic evaluation, assessment of severity, isolation and the need for hospitalisation. Because there is a higher risk of adverse outcomes, patients with diabetes should be preferentially managed in hospitals or settings where close monitoring of disease progression is possible. For those managed at home, regular telephone contact with healthcare services and follow-up is crucial to recognise deterioration in glycaemic control, development of hyperglycaemic emergencies or deterioration of clinical status.

Frequent glucose monitoring, healthy diet, adequate hydration and dose titration of glucose-lowering medication in liaison with healthcare providers should be prioritised. Patients may take symptomatic therapy, including paracetamol/acetaminophen, which is the preferred anti-pyretic agent [45]. However, paracetamol may interfere with the accuracy of certain CGM sensors [46]. In such situations serial capillary blood sugar monitoring can be adopted [47]. The use of non-steroidal anti-inflammatory drugs (NSAIDs), including ibuprofen, for symptom relief has raised several concerns; its suppressive effect on immune response is thought to delay recovery, while salt and water retention could worsen lung injury [48]. NSAIDs are also thought to upregulate ACE2 (see previous section ‘ACE2, SARS-CoV-2 and diabetes’) [39].

#### Glycaemic control

The importance of good glycaemic control during the COVID-19 pandemic is emphasised [49]. As this is a new viral infection, the data are limited and so expert recommendations conform with strategies used in similar epidemics [47]. Patients with mild COVID-19 can be managed with their usual glucose-lowering agents as long as they are able to eat and drink satisfactorily. Patients should adhere to the ‘sick day rules’ for people with diabetes [50], and frequent monitoring of capillary glucose is important. Patients who develop severe illness are likely to need treatment modifications. Multiple factors including glycaemic status, haemodynamic stability, nutritional status, renal function, risk of hypoglycaemia, drug interactions and the availability of medications influence the decision making. There are specific concerns in relation to glucose-lowering agents when used in patients with COVID-19 (Table 1). Metformin may be stopped in patients who are hospitalised and acutely ill, owing to the risk of lactic acidosis [51]. In severe COVID 19 infection, the hypoxic state may further increase the risk of lactic acidosis.

**Table 1:** The use of pharmacotherapies for diabetes and related comorbidities during COVID-19

Therapy	Considerations for use during COVID-19	Suggestions for practice
Metformin	Risk of lactic acidosis in hypoxia and acute illness	Stop if severely ill with haemodynamic instability or hypoxia
SGLT2 inhibitors	Increased risk of dehydration and euglycaemic ketoacidosis	Stop if oral intake is not tolerated or severely ill
GLP-1RAs	Gastrointestinal side effects and risk of aspiration	Stop in severely ill patients
DPP4 inhibitors	Low risk of hypoglycaemia; possible to use for a wide range of renal function	May be continued in non-critically ill patients
Sulfonylureas	Risk of hypoglycaemia if oral intake is poor or with concomitant use of hydroxychloroquine or chloroquine	Stop if unable to maintain regular oral food intake or at risk of hypoglycaemia
Pioglitazone	Risk of fluid retention and oedema; contraindicated in haemodynamic instability	Stop if severely ill with haemodynamic instability, or hepatic or cardiac dysfunction
Insulin	Requires frequent monitoring due to risk of hypoglycaemia	Drug of choice in critically ill patients (see text)
ACEI/ARBs	Uncertain risk of increased susceptibility for infection and uncertain benefit in mitigating inflammatory injury	Continue use unless a specific contraindication arises (hypotension, hyperkalaemia, acute kidney injury)
Aspirin	Risk of cardiovascular disease higher during COVID infection	Continue for patients on aspirin for secondary prevention unless contraindications arise
Statins	Possibility of increased risk of transaminitis and myositis	Individualised decision on risk and benefit

Although there are no specific data on sodium–glucose cotransporter 2 (SGLT2) inhibitor use during COVID-19 infection, it is advisable to withhold these during acute illness because of the increased risk of dehydration and euglycaemic ketoacidosis [52, 53], as well as difficulties in maintaining usual perineal hygiene. Glucagon-like peptide-1 receptor agonist (GLP-1RA) therapy should probably be temporarily discontinued in patients with haemodynamic instability (which compromises absorption from subcutaneous sites), renal dysfunction, and gastrointestinal dysfunction (which prevents adequate oral intake). Treatment with GLP-1RAs may cause gastrointestinal side effects, predisposing to volume depletion and aspiration [54]. Dipeptidyl peptidase-4 (DPP4) inhibitors are associated with low risk of hypoglycaemia and are relatively safe across a wide range of renal functions. DPP4 inhibitors added to basal insulin improve glycaemic control without increasing the risk of hypoglycaemia, even among hospitalised patients [55]. However, these agents are likely to be of less therapeutic benefit in patients with severe COVID-19. While patients with mild symptoms could continue DPP4 inhibitors, these should be omitted in acute severe illness and replaced with insulin if this becomes necessary. During a severe illness, fine control of blood glucose is difficult when using sulfonylureas. In such patients, sulfonylureas should be replaced with insulin. Caution with sulfonylureas also needs to be exercised if chloroquine is considered, because of risk of hypoglycaemia with both. Thiazolidinediones (e.g. pioglitazone) are a less favourable option for in-hospital management of acutely ill patients, because of fluid retention and oedema. They are contraindicated in patients with haemodynamic instability, or hepatic or cardiac dysfunction, which may be seen in severe COVID-19 infection [56].

Existing evidence favours insulin over other glucose-lowering agents for glycaemic control in hospitalised patients. For non-critically ill hospitalised patients, subcutaneous insulin therapy with basal or intermediate-acting insulin given once or twice a day, along with mealtime boluses of short- or rapid-acting insulin, is the preferred strategy for glycaemic management [57]. Sliding scale therapy should probably not be practiced as this results in greater fluctuations and poor overall control [58]. For critically ill patients, insulin therapy should be initiated with a glycaemic target of 7.8–10 mmol/l (140–180 mg/dl) [59]. Less stringent glycaemic control with target glucose concentrations >10 mmol/l (180 mg/dl) may be acceptable in terminally ill patients, in patients with severe comorbidities, and in patient care settings where frequent glucose monitoring or close nursing supervision is not feasible. Less aggressive insulin regimens aimed at simply minimising glucosuria, dehydration and electrolyte disturbances may be justifiable in such patients. Although there are no strict guidelines regarding the insulin regimen for glycaemic control in critically ill patients, intravenous insulin infusions and short or rapid-acting insulin boluses may be used. Disease severity, nutritional status, concomitant medications and trend of glycaemic fluctuations should be considered in determining insulin dosage [60].

In patients with type 1 diabetes with COVID-19 and hyperglycaemia, it is important to monitor the blood glucose and ketone levels, maintain hydration and continue insulin therapy.

Strict infection control measures should be implemented for COVID-19 patients with diabetes irrespective of where they are treated, considering their vulnerability to acquiring secondary bacterial infections, with potential serious consequences.

#### Medical therapy for comorbidities

ACEI/ARBs are essential in management of hypertension, heart failure and diabetic nephropathy. Considering the contrasting effects of ACE2 on CoV infection and inflammatory lung injury (see above and Fig. 1), it is difficult to predict the possible clinical outcomes. To date, no clear evidence exists for or against the use of ACEI/ARBs in people with diabetes at risk or infected with SARS-CoV-2, despite the speculations for potential adverse effects [39]. There are clear competing risks in stopping, since the control of hypertension and the protection against renal disease may be compromised. At present, most international organisations have recommended continuation of ACEI/ARBs, unless there are explicit contraindications such as hypotension or acute kidney injury [32, 61].

There is no clear evidence of risks associated with continuing aspirin. Although myocardial injury is a well-known serious manifestation of COVID-19, acute myocardial ischaemia is not clearly described. Concerns of atherosclerotic plaque accidents and increased acute ischaemic strokes exist [62, 63]. Until further data are available, it would be appropriate to continue aspirin for patients with indication for secondary prevention unless specific individual concerns such as gastrointestinal bleeding are noted.

At present, there is no direct evidence for or against continuation of statins in patients with diabetes and COVID-19**.** There are preliminary reports of raised liver enzymes and muscle enzymes associated with COVID-19 although severe liver disease or rhabdomyolysis are not characteristic [64]. Therefore we suggest an individualised decision considering the indication for statin therapy as well as possible drug interactions with antiviral agents.

### Specific therapies for COVID-19 in people with diabetes

Several authorities have proposed guidelines and protocols for the management of COVID-19 (electronic supplementary material [ESM] Table 1). However, dedicated guidance for management of COVID-19 in people with diabetes has not been formulated. Most guidelines suggest that treatment should be limited to patients with virologically confirmed COVID-19 (ESM Table 1). Although no drug has robust evidence on specific antiviral efficacy or on clinical outcomes in the treatment of COVID-19, several medications are being used on the basis of limited clinical data, or being tested in clinical trials. Safety concerns of candidate agents should be weighed against their relative benefit. There are specific concerns in relation to some of these agents for people with diabetes (Table 2). In situations where compassionate or off label anti-COVID-19 therapy is considered this is best done within systematic clinical trials.

**Table 2:** Concerns for people with diabetes in using proposed therapeutic agents for COVID-19

Therapeutic agent	Considerations for people with diabetes
Chloroquine/ hydroxychloroquine	• Hypoglycaemia: caution with insulin and insulin secretagogues • Prolongation of QT interval: caution in people with comorbid cardiovascular disease. Risk increased by azithromycin
Lopinavir/ritonavir	• Hyperglycaemia, deterioration of glycaemic control • Interaction with statins: increased risk of hepatic and muscle toxicity
Glucocorticoids	• Hyperglycaemia • Susceptibility to secondary bacterial infection
Remdesivir	• Hepatotoxicity: caution with statins and pre-existing fatty liver disease

#### Immunomodulators

Cytokine release syndrome (‘cytokine storm’) is thought to be central to the pathogenesis of rapid deterioration and multi-organ dysfunction in patients with COVID-19. Therefore, immunomodulatory agents are postulated to be of benefit.

*1. Chloroquine/hydroxychloroquine* These two antimalarial agents have attracted much attention in the treatment of COVID-19 on the basis of limited clinical experience, as well as due to political reasons. Chloroquine has shown antiviral and anti-inflammatory properties in previous experimental studies [65]. Hydroxychloroquine has shown superior potency compared with chloroquine in experimental studies [66] and has a more favourable safety profile, probably because it has a lower level of tissue accumulation [67].

A study from France, involving 36 virologically confirmed patients with COVID-19, showed that chloroquine, at a dose of 600 mg a day for 10 days, reduced the virus carriage significantly at 6 days of therapy compared with standard care [68]. However, this was a non-randomised study, recruited less than the estimated sample size, was under-powered for assessment of clinical outcomes, had differences between intervention and control groups (older mean age and higher azithromycin use in intervention group) and a higher dropout rate in the intervention arm (due to 3 ICU admissions, 1 death, 2 withdrawals of consent), emphasising the need for very cautious interpretation.

A systematic review of literature by Cortegiani et al. concluded that evidence is limited to experimental in vitro studies showing antiviral potency of chloroquine/hydroxychloroquine [69]. More than 20 ongoing randomised clinical trials are investigating the utility of these agents (Solidarity trial for hydroxychloroquine and remdesivir [WHO, NCT04321616]; several studies from China [70] and Minnesota University [COVID-19 PEP trial: hydroxychloroquine for post-exposure prophylaxis and pre-emptive therapy of COVID-19, NCT04308668]).

There are specific safety concerns for people with diabetes, as hypoglycaemia is a known adverse effect of chloroquine/hydroxychloroquine treatment. Suggested mechanisms are decreased intra-cellular insulin degradation, increased insulin-mediated glucose transport, increased insulin release and enhanced insulin sensitivity [71]. Therefore, extra caution should be exercised when used with other glucose lowering agents and dose reduction may become necessary.

Prolongation of the QT interval and serious cardiac arrhythmias are known, albeit rare, dose-dependent adverse effects of chloroquine/hydroxychloroquine. The risk is greater in those with pre-existing cardiac disorders, especially comorbid coronary artery disease and diminished cardiac reserve. COVID-19 itself is known to induce myocardial injury, adding to the risk. Regular ECG monitoring before and while on therapy is recommended. Some guidelines recommend against combining with other QT prolonging agents such as azithromycin, lopinavir/ritonavir while others recommend cautious use (ESM Table 1).

*2. Glucocorticoids* Although glucocorticoids are used in the treatment of severe acute respiratory distress syndrome (ARDS), data for their use in ARDS caused by viral pneumonia are minimal and therefore they are not recommended for routine use in COVID-19 [72]. The place of glucocorticoids in the treatment of COVID-19 is being investigated (NCT04273321). If they are used in patients with diabetes, hyperglycaemia may worsen, necessitating escalation of insulin therapy.

#### Antivirals

The FDA has authorised emergency use of remdesivir, which, although not affecting mortality, significantly shortens the course of the disease [73]. Further studies are in progress to evaluate this treatment as well as other antiviral agents (ESM Tables 2 and 3). If proven to be effective among patients without diabetes, it is likely that patients with diabetes would also benefit from such therapeutic agents, although caution should always be exercised in patients with multi-morbidity.

### Glucose-lowering agents as potential therapeutic option for COVID 19

*1. Metformin* Yu et al. showed that metformin reversed lipopolysaccharide-induced pulmonary oedema, vascular leakage and neutrophil accumulation, and reduced the levels of TNF-α, IL-1β, IL-6 and IL-17 in an ARDS model [74]. Patients with severe SARS-CoV-2 develop ARDS, which is mediated by dysregulated immune response producing a cytokine storm. However, hypoxia in severe disease limits its use due to a risk of lactic acidosis. Further research is needed regarding the role of metformin as a host-directed treatment for severe COVID -19 [75].

*2. Incretin based therapies* DPP4 is a ubiquitous type II transmembrane glycoprotein expressed in many cells, including the alveolar epithelium and inflammatory cells. MERS-CoV uses DPP4 to gain entry into host cells [76]. DPP4 inhibition mitigated inflammatory response in experimental studies [77]. It is not known if SARS-CoV-2 uses DPP4 for cell entry. To date, neither benefit nor harm has been shown in humans on DPP4 inhibitors during CoV infections. Therefore, DPP4 inhibitors could be continued, at least in mild cases of COVID-19, while potential benefit in treating CoV infection remains to be studied further [78]. Similarly, GLP-1RAs are known to have anti-inflammatory effects and have shown potential for therapeutic benefit in acute lung injury [79]. However data are limited to experimental models and their benefit, at best, remains speculative.

## Prevention of COVID-19 in people with diabetes

General precautions are mandatory for patients and caregivers, to prevent contracting COVID-19 (Text box: General precautions to prevent COVID-19 in people with diabetes). Chemoprophylaxis (pre- and post-exposure) and vaccines are other strategies under evaluation.

### Chemoprophylaxis

No agent had been approved so far for pre- or post-exposure chemoprophylaxis. Evidence from randomised clinical trials is urgently needed. Chloroquine has demonstrated antiviral activity against five out of seven known human coronaviruses, including COVID-19 [80] and is a leading candidate for prophylactic use [81]. Ongoing trials in China have yielded encouraging preliminary findings [70], but the data are generally contentious. Several other trials are in progress: the PHYDRA Trial (NCT04318015) and COPCOV study (NCT04303507). Patients with diabetes are also included in these studies. A cluster-randomised controlled trial is planned to evaluate the use of lopinavir/ritonavir in post-exposure prophylaxis (NCT04321174).

### Vaccines

A safe and potent vaccine would obviously be very useful for high risk individuals, such as those with diabetes or cardiovascular disease and the elderly. Several vaccines are being investigated: the APICTH trial: recombinant novel coronavirus vaccine (adenovirus type 5 vector) (NCT04313127); mRNA-1273 vaccine (NCT04283461) and artificial antigen-presenting cells (aAPCs) as a vaccine (NCT04299724).

## Future directions

COVID-19 has emerged as one of the greatest challenges for humankind after the Second World War. Identification of effective preventive and treatment strategies is urgently needed. People with diabetes and related comorbidities have been shown to fare worse, although the pathophysiological and molecular mechanisms behind this link are not yet fully understood. Researchers and authorities worldwide should take urgent steps to answer critical questions in the prevention and management of COVID-19 and the protection of people with diabetes (Text box: Unanswered questions).

It is imperative to establish standard case definitions, data collection, recording and sharing strategies and operational guidelines to allow comparison and analysis of data. Standardisation of research protocols and identification of research priorities is essential to utilise time and resources productively. The role of pharmaceutical agents in the prevention and treatment of COVID-19, in terms of their efficacy, safety and cost effectiveness, should be evaluated as a priority. Further data are needed, especially looking at the effects of ACEI/ARBs and SGLT2 inhibitors in those infected, as well as in the severely ill.

Healthcare systems should adopt strategies for case detection and treatment while maintaining care and supply of essential medicines for people with chronic diseases such as diabetes, to reduce morbidity and mortality risk due to such diseases during this period. The strategic utilisation of human resources in healthcare services and safeguarding their health is a timely need. The current challenge for healthcare systems should be an opportunity to improve service provision, learn from successful regional and global strategies and prepare for future challenges of greater magnitude. The pandemic also highlights the need for joined-up public health measures and care-for-all policies.

## Electronic supplementary material

ESM Tables (PDF 238 kb)

Slideset of figures (PPTX 495 kb)

## Abbreviations

ACEI: ACE inhibitor
ARB: Angiotensin receptor blocker
ARDS: Acute respiratory distress syndrome
CGM: Continuous glucose monitoring
CoV: Coronavirus
COVID-19: Coronavirus disease 2019
CRP: C-reactive protein
DKA: Diabetic ketoacidosis
DPP4: Dipeptidyl peptidase-4
ESR: Erythrocyte sedimentation rate
GLP-1RA: Glucagon-like peptide-1 receptor agonist
ICU: Intensive care unit
MERS: Middle East respiratory syndrome
MERS-CoV: Middle East respiratory syndrome-coronavirus
NSAID: Non-steroidal anti-inflammatory drug
SARS: Severe acute respiratory syndrome
SARS-CoV: Severe acute respiratory syndrome-coronavirus (caused 2002/2003 epidemic)
SARS-CoV-2: Severe acute respiratory syndrome-coronavirus-2 (causing current COVID-19 pandemic)
SGLT2: Sodium–glucose cotransporter 2
