# Supplementary material for: Prevention and management of COVID-19 among patients with diabetes: an appraisal of the literature
Source: Diabetologia. 2020 May 14;63(8):1440–52. doi: 10.1007/s00125-020-05164-x (PMC7220850; doi:10.1007/s00125-020-05164-x)
Supplement: Supplementary file 1 — (PDF 238 kb) [file 125_2020_5164_MOESM1_ESM.pdf]

ESM Table 1

Summary of selected guidelines on specific pharmacological management of COVID-19*

Source	When to treat	Mild disease	Severe disease
WHO interim guidelines (12th March 2020)	No evidence to recommend specific therapy	No evidence to recommend specific therapy	No evidence to recommend specific therapy
Center for Disease Control and Prevention (CDC), Atlanta, USA	PCR Confirmed COVID-19	**URTI**:<br>CQ 500 mg BID 5d OR Oseltamivir 150 mg BID 5d	CQ 500 mg BID 5d plus Darunavir 800 mg / Cobicistat 150 mg QD 14d OR<br>• Atazanavir 400 mg QD 14d plus Oseltamivir 150 mg BID 5d
Surviving Sepsis Campaign, (SCCM & ESICM)	Insufficient evidence; no recommendation	Insufficient evidence; no recommendation	Insufficient evidence; no recommendation
China: Expert consensus from Department of Science and Technology and Health Commission of Guangdong province, China	Confirmed COVID-19	**For mild moderate or severe coronavirus pneumonia:**<br>CQ 500 mg BID 10d	
Korea: Central Clinical Task Force	Moderate to severe disease, or elderly or multiple co-morbidities	Supportive care only	**Moderate to severe disease:**<br>Lopinavir 400mg/Ritonavir 100mg BID or<br>CQ 500mg QD or HCQ 400mg QD 7-10d
Italy: Italian Society of Infectious and Tropical Diseases	Confirmed COVID-19	**Mild to moderate**:<br>Lopinavir/ritonavir plus CQ 500 mg BID OR<br>HCQ 200 mg QD 10d	**Severe or critical**:<br>Remdesivir plus CQ 500 mg BID OR<br>HCQ 200 mg QD 10-20d
Belgium: Interim clinical guidelines	Consider in confirmed or highly suspicious COVID-19 patients.	**Mild / moderate / severe**:<br>HCQ 400mg BID 2 doses, then 200mg BID 5d OR<br>CQ 600mg stat and 300mg BID 5d<br>If above are contraindicated:<br>lopinavir 400 mg / ritonavir 100 mg BID	**Critically ill**:<br>Remdesivir IV 200 mg loading dose over 30 min followed by IV 100 mg QD for 2-10d

		14d, starting within 10d of symptom onset	

*All drugs are administered orally unless otherwise specified. (Last update 26th March 2020)
AZT: Azithromycin, BID: 12 hourly, CDC: Center for Disease Control and Prevention, CQ: chloroquine, d: days, ESICM: European Society of Intensive and Critical Care Medicine, HCQ: hydroxychloroquine, QD: ever 24 hours, SCCM: Society of Critical Care Medicine, URTI: upper respiratory tract infection, WHO: World health Organization

ESM Table 2

Candidate drugs for the treatment of COVID-19

Agent and class	Mechanism of action	Concerns for use in people with diabetes	Other adverse effects
Chloroquine (4-aminoquinoline)	Not certain. Possible multiple mechanisms:<br>- Inhibit viral entry to host cells by interfering glycosylation of ACE2<br>- Inhibit replication by inhibiting MAP-kinase<br>- Inhibit cell entry by inhibiting quinone reductase- 2 which is necessary for sialic acid biosynthesis (act as receptor)<br>- Alters cell pH, thus inhibiting viral entry, transport & post entry events	Improves glycaemic control,<br><br>Risk of hypoglycaemia	Gastrointestinal disturbance<br><br>QT prolongation – monitor ECG daily, avoid in recent MI. Risk increased when co-prescribed with azithromycin, teneligliptin, lopinavir / ritonavir and remdesivir<br><br>Cytopaenia<br><br>Retinopathy (with long term use), maculopathy<br><br>Vertigo, tinnitus, deafness<br><br>Cardiomyopathy, heart failure (rare)<br><br>Avoid in G6PD deficiency, porphyria, liver failure, myasthenia gravis, epilepsy
Hydroxychloroquine (4-aminoquinoline) *The structure is similar to CQ except the addition of hydroxyl moiety in a terminal	Same as CQ	Same as CQ	Same as CQ. Retinopathy less common.
Remdesivir (adenosine nucleotide analogues)	Inhibits viral replication, probably by inhibiting	No significant effect	Limited data

	RNA dependent RNA polymerase		
Lopinavir/ Ritonavir (Protease inhibitors)	Inhibits formation of infectious virions	New onset diabetes mellitus, exacerbation of pre-existing diabetes mellitus, and hyperglycemia	Caution: multiple interactions
Azithromycin (macrolide)	To treat secondary bacterial pneumonia with its antibacterial action.<br>Binds to the 50s subunit of the bacterial ribosome, thus inhibiting translation of mRNA.<br>Possible anti-inflammatory effect	No significant effect	Abnormal liver function tests, long QT (caution with CQ, HCQ), pseudomembranous colitis, DRESS syndrome

ECG: electrocardiogram, CQ: chloroquine, HCQ: hydroxychloroquine, G6PD: glucose 6 phosphate dehydrogenase, QT: QT interval, DRESS: Drug reaction with eosinophilia and systemic symptoms, ACE2: angiotensin converting enzyme

ESM Table 3

Other investigational antiviral and immunomodulatory agents for treatment of COVID-19 (updated on 29th March 2020, www.clinicaltrials.gov)

Drug	Class	Mechanisms of action
**Antivirals**		
Ribavirin	Nucleoside analogue	Inhibits viral RNA synthesis and mRNA capping
Darunavir/Cobicistat	Protease inhibitors	Blocks viral cellular entry
SARS-Cov-2 specific protease drug candidate		
Favipiravir	RNA polymerase inhibitors	Inhibits viral RNA dependent polymerase
Oseltamivir	Neuraminidase inhibitor	Inhibits viral replication
Baloxivir marboxil	Viral endonuclease inhibitor	Inhibits influenza virus multiplication
Umifenovir	Fusion inhibitor	Inhibits fusion between viral and cellular membrane
Carrimycin	Macrolide	Not certain
Arbidol	Fusion inhibitor	Broad spectrum antiviral
**Immunomodulators**		
Non-specific, broad spectrum		
Colchicine	Anti-mitotic agent	Inhibit immune cell proliferation
Methylprednisolone	Steroidal anti-inflammatory agent	Anti-inflammatory
Tetrandrine	Calcium channel blocker	Anti-inflammatory and immunomodulatory
Fingolimod	Lymphocyte inhibitor	Anti-inflammatory
Thalidomide	Inhibit IL-6 production, other mechanisms	Anti-inflammatory
Xiyanping	Andrographolide – immunomodulator	Immunomodulatory
Aviptadil	VIP analogue	Anti-inflammatory
Targeted immunomodulators		
Interferon-ß1A, interferon alpha 1ß, interferon-ß-1B	Cytokines	Stimulate innate antiviral immunity
Aerosolized interferon α		
Tocilizumab, Sarilumab	Anti-IL-6 monoclonal antibody	Inhibits IL-6 mediated inflammatory cascade
Eculizumab	Anti-C5 monoclonal antibody	Inhibit complement activation and inflammation

SARS-Cov-2 specific antibodies	Antibody	Binds to virus and block infection, binds to infected cells and change the immune system
Bevacizumab	Anti-VEGF-A monoclonal antibody	Inhibits angiogenesis, anti-inflammatory
Camostat mesylate	Serine protease inhibitor	Immunomodulatory
Baricitinib	Janus kinase inhibitor	Immunomodulatory
aAPC	Activated APCs as immunomodulators	Facilitate immune response against infected cells
NK cells	Natural killer cells as immunomodulators	Facilitate immune response against infected cells
**Combinations**		
Ribavirin plus Interferon	Nucleoside analogue plus cytokine	Inhibits viral replication and stimulate innate immunity
Interferon beta plus Lopinavir/Ritonavir	Cytokine plus protease inhibitor	stimulate innate antiviral immunity and blocks viral cellular entry

APC: antigen presenting cells, IL-6: interleukin-6. NK cells: natural killer cells, VEGF-A: vascular endothelial growth factor-A
