# Supplementary material for: Prevention and management of COVID-19 among patients with diabetes: an appraisal of the literature
Source: Diabetologia. 2020 May 14;63(8):1440–52. doi: 10.1007/s00125-020-05164-x (PMC7220850; doi:10.1007/s00125-020-05164-x)
Supplement: Supplementary file 2 — (PPTX 495 kb) [file 125_2020_5164_MOESM2_ESM.pptx]

## Slide 1
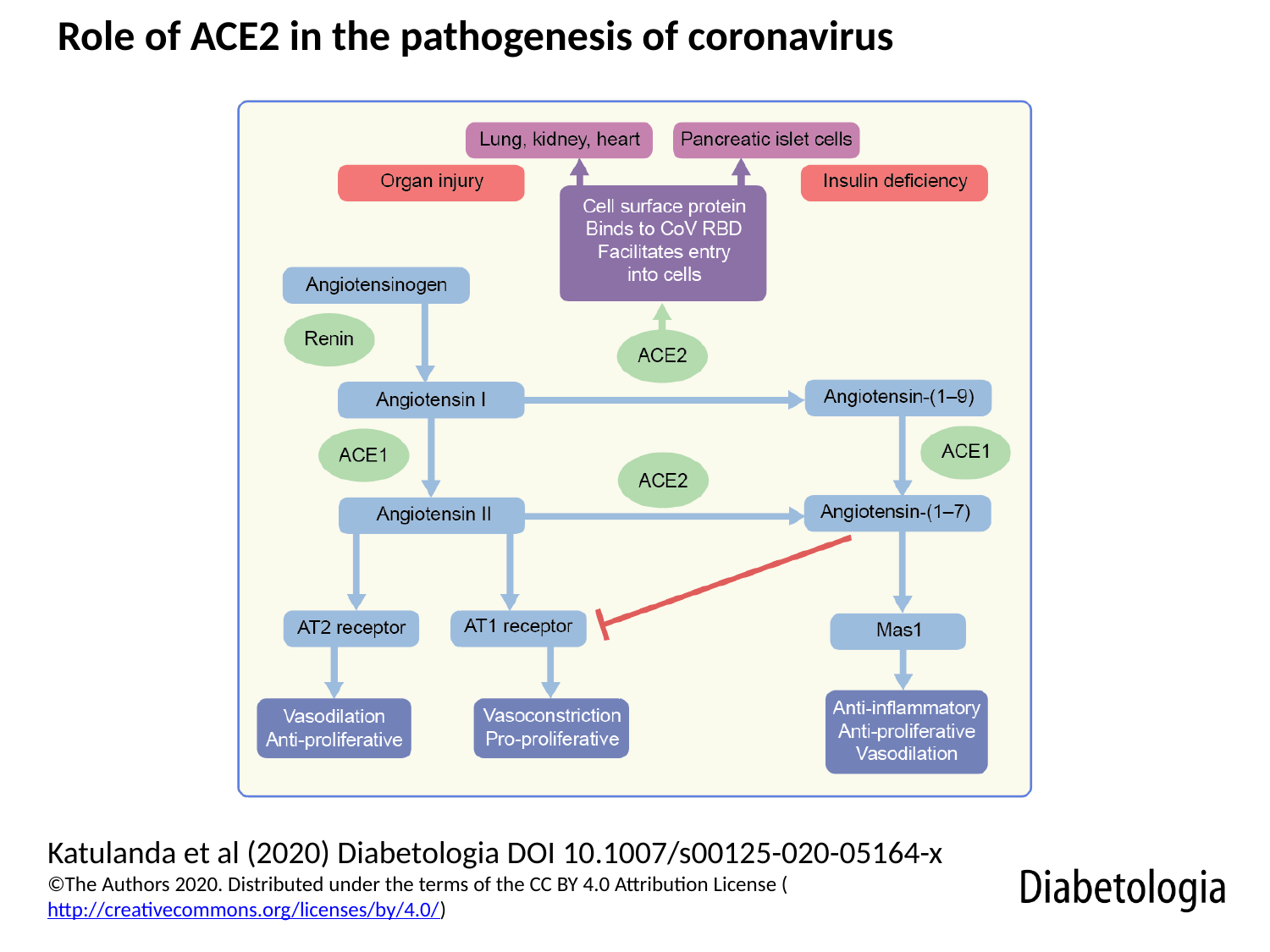

Role of ACE2 in the pathogenesis of coronavirus
Katulanda et al (2020) Diabetologia DOI 10.1007/s00125-020-05164-x
©The Authors 2020. Distributed under the terms of the CC BY 4.0 Attribution License (http://creativecommons.org/licenses/by/4.0/)

## Slide 2
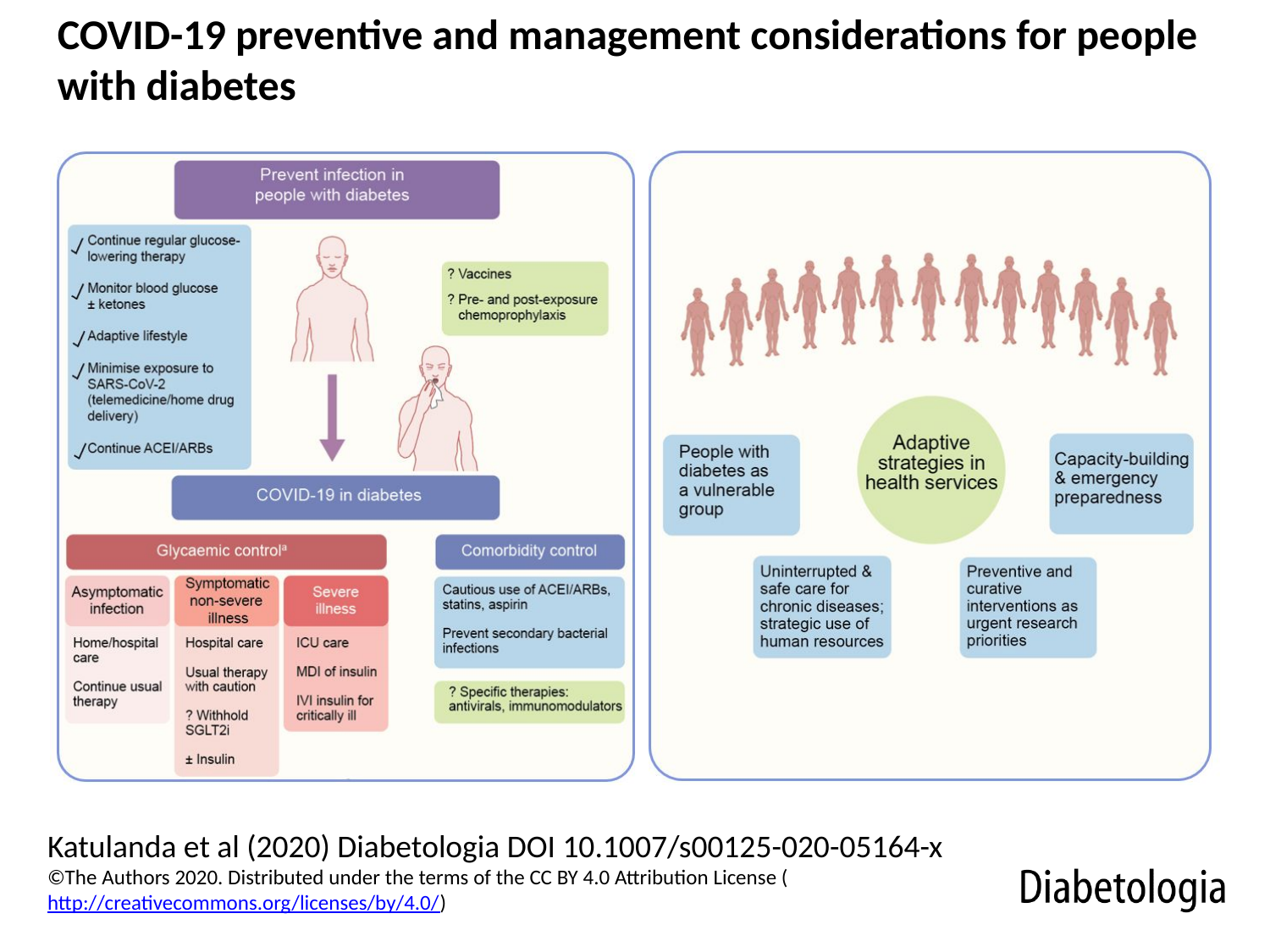

COVID-19 preventive and management considerations for people with diabetes
Katulanda et al (2020) Diabetologia DOI 10.1007/s00125-020-05164-x
©The Authors 2020. Distributed under the terms of the CC BY 4.0 Attribution License (http://creativecommons.org/licenses/by/4.0/)
